# Do we need a Unique Scientist ID for publications in biomedicine?

**DOI:** 10.1186/1742-5581-2-1

**Published:** 2005-03-22

**Authors:** Andreas Bohne-Lang, Elke Lang

**Affiliations:** 1 German Cancer Research Center Heidelberg, Central Spectroscopy – Molecular Modeling, Im Neuenheimer Feld 280, 69120 Heidelberg, Germany; 2 University of Applied Sciences Darmstadt, Information and Knowledge Management, Campus Dieburg, Max-Planck-Strasse 2, 64807 Dieburg, Germany

## Abstract

**Background:**

The PubMed database contains nearly 15 million references from more than 4,800 biomedical journals. In general, authors of scientific articles are addressed by their last name and forename initial.

**Discussion:**

In general, names can be too common and not unique enough to be search criteria. Today, Ph.D. students, other researchers and women publish scientific work. A person may not only have one name but several names and publish under each name. A Unique Scientist ID could help to address people in peer-to-peer (P2P) networks. As a starting point, perhaps PubMed could generate and manage such a scientist ID.

**Summary:**

A Unique Scientist ID would improve knowledge management in science. Unfortunately in some of the publications, and then within the online databases, only one letter abbreviates the author's forename. A common name with only one initial could retrieve pertinent citations, but include many *false drops *(retrieval matching searched criteria but indisputably irrelevant).

## Background

**Figure 1:**
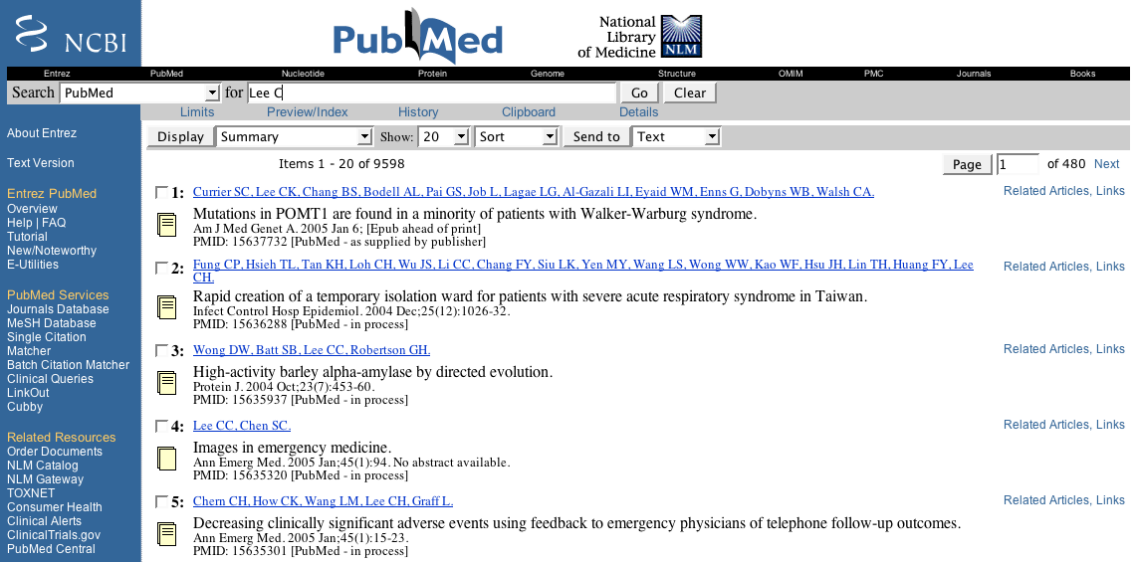
**PubMed search for 'Lee C'**. A simple search for 'Lee C' results over 9000 articles from PubMed.

The National Library of Medicine (NLM) created PubMed, which is one of the largest literature databases in biomedicine. The database contains nearly 15 million references from more than 4,800 biomedical journals. In general, authors of scientific articles are addressed by their last name and forename initial. For example, a search for "Lee C", a common Chinese name, would retrieve over 9000 hits (Fig 1).

## Discussion

In general, names can be too common and not unique enough to be search criteria. Modern life advances this point. In former times only a few scientists, normally professors in universities and academic health sciences centers, published scientific results of their work under their names. Most scientists were men. Their names were consistent and did not change by marriage. Today, Ph.D. students, other researchers and women publish scientific work. Women may be using a married name, which may be a compound name for authorship. Thus a person may not only have one name but several names and publish under each name. In Germany, there can be nameconfusion for titles of nobility from former times or for composed names. Claus-Wilhelm von der Lieth (with 'Claus-Wilhelm' as forename and 'von   der Lieth' as surname) is cited in several mutations, such as   'Vonderlieth CW', 'Lieth von der CW' or only 'Lieth CW'.   

Especially with a view to the future, such a Unique Scientist ID could help to address people in peer-to-peer (P2P) networks. One such network based article reference P2P network is Bibster [1]. Bibster is a Java-based system which assists researchers in managing, searching and sharing bibliographic metadata in a peer-to-peer network.

How can one easily find articles written by a particular person? A Unique Scientist ID could help to address persons. A Unique Scientist ID should contain all the versions of a scientist's name. As a starting point, perhaps PubMed could generate and manage such a scientist ID. Adding the Scientist ID should become as routine as adding an email address to the article's citation.

## Conclusion

A Unique Scientist ID would improve knowledge management in science. At the moment authors of publications are only addressed in most online databases by name, initials and address. Unfortunately in some of the publications, and then within the online databases, only one letter abbreviates the author's forename. A search for a special author is successful if the name is quite unique or a combination of authors is used for the search. In such a case, a common name with only one initial could retrieve pertinent citations, but include many *false drops *(retrieval matching searched criteria but indisputably irrelevant).

## Authors' contributions

Both authors contributed equally.

## Competing interests

The author(s) declare that they have no competing interests.
